# Electrodegradation of 2,4-dichlorophenoxyacetic acid herbicide from aqueous solution using three-dimensional electrode reactor with G/β-PbO_2_ anode: Taguchi optimization and degradation mechanism determination†

**DOI:** 10.1039/c8ra08471h

**Published:** 2018-11-26

**Authors:** Abdollah Dargahi, Davood Nematollahi, Ghorban Asgari, Reza Shokoohi, Amin Ansari, Mohammad Reza Samarghandi

**Affiliations:** Department of Environmental Health Engineering, School of Health, Hamadan University of Medical Sciences Hamadan Iran a.dargahi29@yahoo.com; Faculty of Chemistry, Bu-Ali-Sina University Hamadan Iran; Research Center for Health Sciences and Dep. Environmental Engineering School of Public Health, Hamadan University of Medical Sciences Hamadan Iran samarghandi@umsha.ac.ir

## Abstract

This study aimed to investigate the electro-degradation of 2,4-dichlorophenoxyacetic acid (2,4-D) from aqueous solution using two and three-dimensional electrode (2D and 3D) reactors with graphite(G)/β-PbO_2_ anode. To increase the degradation efficiency, affecting parameters on the electro-degradation process were investigated and optimized by adopting the Taguchi design of experiments approach. The structure, morphology and electrochemical properties of the electrodes were studied by X-ray diffraction (XRD), scanning electron microscopy (SEM), energy dispersive X-ray spectroscopy (EDX), linear sweep voltammetry and cyclic voltammograms. The controllable factors, *i.e.*, electrolysis time, 2,4-D initial concentration, solution pH and current density (*j*) were optimized. Under optimum conditions, the 2,4-D degradation efficiency was 75.6% using 2D and 93.5% using 3D electrode processes. The percentage contribution of each controllable factor was also determined. The pH of the solution was identified as the most influential factor, and its percentage contribution value was up to 39.9% and 40.4% for 2D and 3D electrode processes, respectively. Considering the parameters of the kinetics, it was found that the degradation of 2,4-D and removal of COD using the G/β-PbO_2_ electrode obey the pseudo-first order kinetics. In addition, the mineralization pathway of 2,4-D at G/β-PbO_2_ electrode was proposed. The results also demonstrated that the 3D electrode process with G/β-PbO_2_ anode can be considered as a useful method for degradation and mineralization of 2,4-D herbicides from aqueous solution.

## Abstract 2

Optimization of process parameters using the Taguchi method, electrochemical degradation and electrochemical degradation mechanism of 2,4-D herbicide using 2D and 3D reactors with G/β-PbO_2_ anode were investigated.

## Graphical abstract

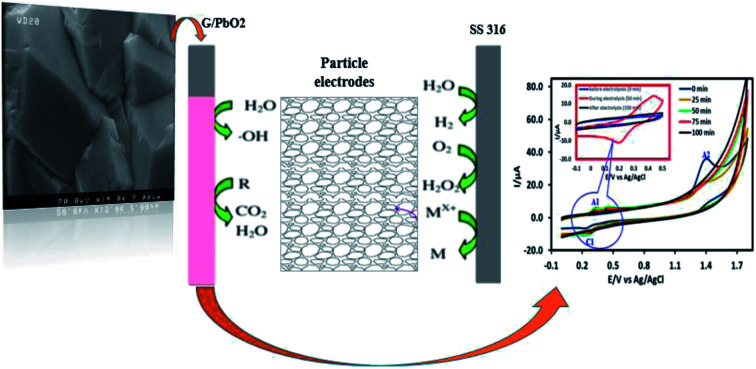

## 1. Introduction

In recent years, the contamination of surface and groundwater by pesticides has become a serious environmental problem due to the extensive use of these chemicals in farmland, gardens and forest lands. Considering their extensive application, the human exposure to such pesticides is almost predictable, which may lead to detrimental effects on human health, including carcinogenicity and mutagenicity.^1^ Pesticides are categorized as persistent organic pollutants and are an integral part of the wastewater generated through the industries producing the fertilizers and the drainage of agricultural activities. Because of this, water sources can be contaminated by various pathways.^2^

The term pesticide is a generic name, which includes a number of the biologically-active compounds, *e.g.*, herbicides, fungicides and insecticides. It has been clarified that there are more than 1400 active ingredients in the various commercial mixtures of pesticides.^3,4^

2,4-Dichlorophenoxyacetic acid, 2,4-D, is known as one of the oldest and most widely used herbicides in the world, its application dating back to 1945, and it is applied for the selective control of weeds in gardens and farms. Its commercial formula contains esters, acids and several salts that are characteristically different and highly toxic.^3,5^ The chemical structure of 2,4-D is presented in Fig. S1.†

Based on the insufficient evidence in humans and limited evidence in experimental animals, the International Agency for Research on Cancer of the World Health Organization has categorized the 2,4-D as “possibly carcinogenic substance to humans”.^2,5,6^ The extensive use of pesticides, and inappropriate wastewater treatment methods has led the contamination of water resources, and the creation of hazardous effects on ecology and the environment.^6^ Hence, treating the wastewater prior to releasing it into the environment is necessary.

Accordingly, a number of the techniques, *e.g.*, advanced oxidation process (AOPs),^3,7,8^ adsorption,^2,9^ plasma-ozonation,^10^ photo-catalytic degradation,^11–13^ electrochemical process,^14^ have been immeasurably applied to remove the 2,4-D herbicide; but, these techniques were not sufficiently successful and were associated with serious problems such as high cost, incomplete pollutant removal, production of toxic and additional toxic products, the need for adding the chemical compounds, sludge production and need for more treatment.^11^ One of the new techniques to treat the water and wastewater is the electrochemical advanced oxidation processes (EAOPs), which are considered as the eco-friendly method due to the application of non-toxic reagent, *i.e.*, the electron, in these processes. The simplest and commonly used EAOP is anodic oxidation or electrochemical oxidation in which the physisorbed *M*(·OH) radical formed by the water oxidation participates to degrade the organics.^15^

After several decades of development, a rapid and profound progress has been observed in context of the electrochemical oxidation processes, especially on anode research. The anode electrodes, due to having a great potential for the oxygen evolution reaction (OER) and having the capability to produce the weakly adsorbed hydroxyl radicals, are as accounted for as the superb anodes for electro-oxidation of the organic pollutants.^14^ Hereupon, these electrodes are divided into two groups, *i.e.*, active electrodes (RuO_2_, IrO_2_, Pt) and non-active electrode (PbO_2_, TiO_2_, SnO_2_, BDD).^16^ The oxygen evolution over potential of non-active electrodes are remarkable and oxidative degradation using these electrodes is directly performed by the adsorbed hydroxyl radicals, produced from water discharge.^16–18^ Low cost, ease of preparation, high conductivity, good corrosion resistance, high oxygen evolution potential and long service lifetime has identified as the superior properties of non-active electrodes, *e.g.*, PbO_2_ rather than the active electrodes, *e.g.* platinum or ruthenium oxide.^19^ Moreover, they have found to be stable both at high potentials and at media with different pH values.^20,21^ Zhou *et al.* (2007) studied the influence of electrochemical methods on PbO_2_ and confirmed that the lead dioxide, formed by the galvanostatic method, is more effective and has a continuous structure which shows a low charge transfer resistance. In other studies, the influence of pH media deposition/dissolution of PbO_2_ on BDD support was emphasized. They found that strong alkaline electrolyte can conduct to the dissolution of PbO_2_.^22^ However, in an acidic and neutral media, PbO_2_ morphology has a good correlation with the obtainment of high oxygen over potential.^23^ For a further enhancement of the electrocatalytic proprieties of PbO_2_ during various anodic reactions, the incorporation of metal oxide, such as TiO_2_,^24^ RuO_2_,^25^ CeO_2_,^26^ SS/PbO_2_,^23^ and ZrO_2_,^27^ into lead dioxide matrix was realized. These researches have indicated that the performance of PbO_2_ electrode is influenced significantly by metal oxide particles.^28^

Despite these unique benefits, there are still limits for their industrial application which include the short lifetime of electrode materials and low current efficiency. In addition, mass transfer limitation, small space-time yield, low area–volume ratio and increasing the temperature during the process are of other natural drawbacks of these processes.^29^

These limitations can be reduced by applying the three-dimensional (3D) electrochemical process. The elimination of the target contaminant using the 3D electrode process is much higher due to the higher specific surface area and, consequently, greater active sites compared to the two-dimensional (2D) electrodes.^29^ The 3D electrode process has many similarities to the 2D counterpart in electrode materials and treatment processes, but it differs in terms of the presence of a third electrode. The third electrode, which is also known as a particle or bed electrode, essentially contains a granular or a particle material that fills between two electrodes. At an appropriate voltage, these particles are polarized and a large number of charged microelectrodes are formed in which one of their surfaces acts as an anode and other acts as a cathode. Hence, due to the presence of particle electrodes, the 3D electrode process provides better efficiency than the 2D electrodes.^29^

The exceptional features of particle electrode materials (such as activated carbon, carbon aerogel, and graphite particles), *e.g.* large specific surface area and high electro-activity, provide the greater mass transfer and reduce the energy consumption^29,30^

Among different materials used as particle electrodes, the granular activated carbon (GAC) is most widely used due to its unique properties, such as low cost, chemical stability and high surface area.^31^ In this research, the third dimension consists of the granules of activated carbon and the anode electrode of G/β-PbO_2_. These electrodes are widely used because of the advantages, *e.g.*, easier preparation by electrochemical coatings, low electrical resistance, low cost, availability and good electrochemical activity.^17,20,21^ The mechanism of this electrochemical process is anodic oxidation; the anodic oxidation is one of the most important electrochemical processes for the removal of organic contaminants from water.

In the present study, the reaction mechanisms of 2,4-D herbicide degradation by a three-dimensional electrode reactor with activated carbon, as particle electrodes, was investigated. To develop the use of β-PbO_2_ electrode for mineralization of 2,4-D herbicide, in the present study, the electrocatalytic activity and efficiency of graphite (G)/β-PbO_2_ electrode for 2,4-D herbicide degradation in aqueous solution were studied. The surface morphology and crystal structure of G/β-PbO_2_ electrode were characterized by SEM, EDX and XRD respectively. Furthermore, the behavior of 2,4-D and produced intermediates during oxidative degradation were studied by cyclic voltammetry. This study proposed the mechanisms for the electrochemical oxidation and oxidative degradation of 2,4-D herbicide. The optimization of the 3D electrode and traditional 2D electrolysis process is essential. Taguchi method, as a strong design approach, was adopted to find the optimum operational parameters for achieving the highest 2,4-D degradation efficiency using 3D process in the aqueous solution. In this study, different parameters, *e.g.*, initial pH, initial 2,4-D concentrations, current density (*j*) and electrolysis time were assessed as the controllable factors. Hereupon, the percentage contribution of each abovementioned experimental parameter is determined using the Taguchi method.

## 2. Materials and methods

The parts of materials and methods section including chemicals, preparation of the G/β-PbO_2_ electrode and optimum concentration of the Supporting Electrolyte (SE) dose, can be found at ESI.†

### 2.1. Analysis procedures

A direct current (DC) power supply (DAZHENG PS-305D, China) with the electric current of 0–5 A and voltage of 0–30 V was used to supply the electrical current.

The residues of 2,4-D in the solution, after electrolysis, were measured using a UV-vis spectrophotometer (DR 6000, HACH, USA) and the accuracy and the validity of the observed data was estimated using the high performance liquid chromatography (HPLC) Agilent 1260 infinity (Agilent Technologies Co. Ltd., USA). The conditions to apply the HPLC were as follows: the mobile phase = a mixture of water and acetonitrile (50 : 50 v/v, HPLC grade, Merck), flow rate = 1 mL min^−1^, temperature = 25 °C. The ICP-OES (Optima-8300) was utilized to measure the leaching of Pb^2+^ after the complete degradation of the 2,4-D in the studied processes.

Moreover, the Chemical Oxygen Demand (COD) was determined by COD ampoules (HACH Chemical) using a spectrophotometer (DR 6000, HACH, USA) to assess the mineralization of the 2,4-D herbicide in solution; the accuracy and validity of the COD measurements was assessed by the potassium hydrogen phthalate (KHP).

Linear sweep voltammetry (LSV) and cyclic voltammetry (CV) were implemented using the Autolab PGSTAT-20 instrument monitored with the Electrochemical System Software (Nova) at room temperature. A glassy carbon disc (1.8 mm^2^ area) was the electrode used in the voltammetry experiments. A platinum wire and an Ag/AgCl (3 M) were applied as the counter electrode and the reference electrode, respectively.

The intermediates analysis carried out using the LC/MS (Shimadzu LCMS 2010 A) system equipped with C18 column (100 mm × 2.1 mm) and an electron spray ionization source. The mobile phase consisted of 60/40% acetonitrile/ultra-pure water + 0.1% formic acid was employed for the analysis. Mass spectra (MS) was carried out under following conditions: Mode, ESI^+^; detection gain, 1.8 kV; prob volt, 4 kV; CDL volt, 25 V; gas nebulizer, N_2_ (grade 5); flow gas, 1.2 L min^−1^; CDL temperature, 250 °C; block temperature, 250 °C.

The scanning electron microscopy (SEM) coupled with energy dispersive X-ray spectroscopy (EDX) (model HITACHI S-4160, Japan) was applied to study the surface morphology of the PbO_2_ deposited onto the graphite bed. The analysis of the phase structure of the PbO_2_ layer was carried out using the X-ray diffraction (XRD pattern) by X'Pert Pro diffractometer (Rigaku RINT2200, Japan). The diffractograms were recorded with a 2*θ* step width of 0.1° and a scan rate of 1520 s/0.1° at 40 kV and the electron probe current of 40 mA.

### 2.2. Electrochemical cell

In the present study, the electrochemical degradation of the 2,4-D herbicide was assessed in a cubical Plexiglass reactor, which its working volume was 250 cc (Fig. S2†). In this reactor, the G/β-PbO2 electrode (7.0 × 5.8 × 0.5 cm) was used as the anode and SS 316 (7.0 × 5.8 × 0.1 cm) as a cathode (the distance between these electrodes was 4 cm). To provide the third dimension of the 3D electrode, the space between the two electrodes was filled by the granular activated carbon (GACs) with a constant concentration of 40 g L^−1^. Due to the special characteristics of the reactor, the granular activated carbon was floated between the two electrodes. It should be noted that the two-dimensional electrode is without GACs. In order to obtain the desired potential, sodium sulfate (Na_2_SO_4_) was used as supporting electrolyte.

The preparation of the simulative wastewater was carried out by dissolving 50 100 150 and 200 mg L^−1^ of 2,4-D herbicide in distilled water with 4 g L^−1^ Na_2_SO_4_ as supporting electrolyte. A direct current (DC) power supply (DAZHENG PS-305D, China) with the electric current of 0–5 A and voltage of 0–30 V was applied to supply the electrical current. The magnetic stirrer and the magnet rotor were used to ensure the mixing effects. To eradicate the effect of the 2,4-D herbicide adsorption, the particle electrodes were initially immersed in 2,4-D herbicide simulated wastewater until the saturation and then were utilized to fill between the main electrodes. All batch experiments were performed in duplicate at room temperature.

The measurement of linear sweep voltammetry (LSV) was performed for the studied β-PbO_2_ electrodes on a classical undivided cell configuration with a platinum wire counter electrode and a glassy carbon disc as the working electrode *versus* an Ag/AgCl reference electrode during the electrolysis. Before each experiment, the glassy carbon electrode was carefully polished using the Struers Water Proof Silicon Carbide Paper (granulometry 2400 and 4000) to reach the stable background voltammogram (Na_2_SO_4_ 0.1 M). During the studied process, the degradation of 2,4-D, the formation of the intermediates and the removal of COD were also controlled by LSV. In order to analyze the COD, the samples were digested in the COD reactor for 2 h at 150 °C.

### 2.3. Designation and optimization of electro-degradation experiment

To investigate the electrochemical process efficiency, four parameters, which are effective on the degradation process of 2,4-D herbicides, including pH (3–10), initial concentration of 2,4-D (50–200 mg L^−1^), current density (3–9 mA cm^−2^) and electrolysis time (30–100 min) were selected as the main parameters which each parameter has four levels. The selected levels of each parameter are shown as Taguchi model data in Table S1.† After designing the experiments using the Taguchi method for the four parameters, 16 proposed test steps were determined by the L-16 design. The details of each experiment are presented in Table 1. All of the experiments were repeated twice and entered into the model and then analyzed by the model.

**Table 1:** The condition for Taguchi design experiments and the results obtained for each experiment and their related S/N values^a^

Tests	Factor				HDE (%)				S/N	
	*A*	*B*	*C*	*D*	EC, 2D		EC, 3D		EC, 2D	EC, 3D
					HDE_2_	HDE_1_	HDE_2_	HDE_1_		
Tests 1	3	30	50	3	34.96	33.5	55.35	55.15	30.68	34.84
Tests 2		50	100	5	51.92	49.5	72.31	71.15	34.09	37.11
Tests 3		80	150	7	63.76	62	84.5	82.65	35.96	38.44
Tests 4		**100**	**200**	**9**	**72.71**	**71.2**	**91.87**	**92.23**	**37.14**	**39.28**
Tests 5	5	30	100	7	42.75	43.7	63.14	64.05	32.71	36.06
Tests 6		50	50	9	67.72	66.32	88.44	86.77	36.52	38.85
Tests 7		80	200	3	51.8	51.25	72.88	72.19	34.24	37.21
Tests 8		100	150	5	62.96	61.5	82.65	83.95	35.87	38.41
Tests 9	7	30	150	9	37.3	35.5	57.69	56.15	31.21	35.10
Tests 10		50	200	7	34.9	35.68	56.36	55.73	30.95	34.97
Tests 11		80	50	5	41.14	42.8	61.53	63.75	32.45	35.93
Tests 12		100	100	3	43.26	44.65	63.65	65.82	32.85	36.22
Tests 13	10	30	200	5	11.365	11.5	31.76	32.25	21.16	30.10
Tests 14		50	150	3	19.6	20.15	39.19	41.8	25.96	32.13
Tests 15		80	100	9	51.7	52.42	72.59	72.81	34.33	37.23
Tests 16		100	50	7	47.4	46.83	67.97	67.58	33.46	36.62

a HDE_1_ and HDE_2_ for both electrochemical process represents the herbicide degradation efficiency at first and second test pieces, respectively. The boldfaces correspond to the maximum value of S/N ratio among the 16 tests.

After adjusting all components of the daily prepared solution, the samples were collected at predetermined time intervals by passing through 0.45 μm membrane filter, and the concentration of 2,4-D was determined using a UV-vis spectrophotometer at a wavelength of 282 nm. In addition, the HPLC (Agilent Technologies Co. Ltd., USA) at a wavelength of 282 nm was used to validate the accuracy of the results obtained by the UV-vis spectrophotometer. To assess the mineralization degree, the chemical oxygen demand (COD) was measured based on the standard method.

The eqn (1) was employed to calculate the herbicide degradation efficiency (HDE):1
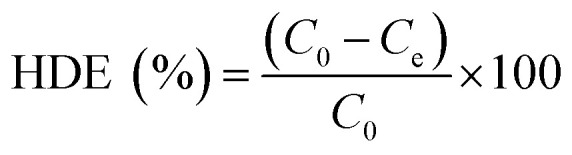
where HDE (%) is herbicide degradation efficiency, *C*_0_ and *C*_e_ (mg L^−1^) are initial concentration and final concentration of 2,4-D herbicide, respectively.

The COD removal was calculated by eqn (2):2
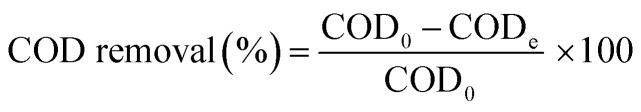
where COD_0_ and COD_e_ represent the COD before and after electrolysis, respectively. The COD removal evolution was measured at the electrolysis time (10, 20, 35, 50, 65, 80 and 100 min).

In the Taguchi method, for the accurate analysis of the results, a converted response function which is defined as the ratio of the sign of each effect (S) to the effects caused by the error (N) is used. In this study, the response is HDE. The S/N ratio is calculated using the eqn (3).^32,33^ The *n* represents the number of replicates of the experiment and HDE shows the results of the experiments. The average S/N ratio for both 2D and 3D electrochemical processes is presented for the analysis of 2,4-D herbicide in Tables 2 and 3.3
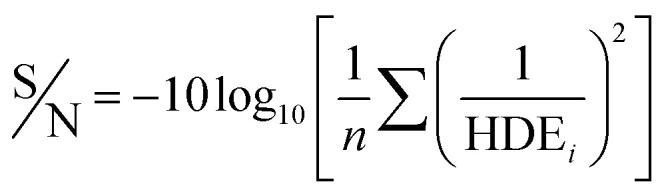


**Table 2:** Results of ANOM Analysis for determination of the optimal conditions for (2D) ED^a^

Factor/level	*j* = 1	*j* = 2	*j* = 3	*j* = 4	*M*
pH/1	30.68	34.09	35.97	37.14	34.47
pH/2	32.71	36.52	34.24	35.88	**34.84**
pH/3	31.21	30.95	32.45	32.85	31.86
pH/4	21.16	25.96	34.33	33.46	28.73
Electrolysis time/1	30.68	32.71	31.21	21.16	28.94
Electrolysis time/2	34.09	36.52	30.95	25.96	31.88
Electrolysis time/3	35.96	34.24	32.45	34.33	34.24
Electrolysis time/4	37.14	35.87	32.85	33.46	**34.83**
2,4-D concentration/1	30.68	36.52	32.45	33.46	33.28
2,4-D concentration/2	34.09	32.71	32.85	34.33	**33.49**
2,4-D concentration/3	35.97	35.87	31.21	25.96	32.25
2,4-D concentration/4	37.14	34.24	30.95	21.16	30.87
Current density/1	30.68	34.24	32.85	25.96	30.93
Current density/2	34.09	35.88	32.45	21.16	30.89
Current density/3	35.97	32.71	30.95	33.46	33.27
Current density/4	37.14	36.52	31.217	31.26	**34.03**

a The boldface corresponds to the maximum value of the mean of the S/N ratios of a certain factor among the four levels.

**Table 3:** Results of ANOM Analysis for determination of the optimal conditions for (3D) ED^a^

Factor/level	*j* = 1	*j* = 2	*j* = 3	*j* = 4	*M*
pH/1	34.85	37.11	38.44	39.28	37.42
pH/2	36.07	38.85	37.21	38.41	**37.63**
pH/3	35.10	34.97	35.93	36.22	35.55
pH/4	30.10	32.13	37.23	36.62	34.02
Electrolysis time/1	34.85	36.07	35.10	30.10	34.03
Electrolysis time/2	37.11	38.85	34.97	32.13	35.77
Electrolysis time/3	38.44	37.21	35.93	37.23	37.20
Electrolysis time/4	39.28	38.4	36.22	36.62	**37.63**
2,4-D concentration/1	34.85	38.85	35.93	36.62	36.56
2,4-D concentration/2	37.11	36.07	36.22	37.23	**36.66**
2,4-D concentration/3	38.44	38.41	35.10	32.13	36.02
2,4-D concentration/4	39.28	37.21	34.97	30.10	35.39
Current density/1	34.85	37.21	36.22	32.13	35.10
Current density/2	37.11	38.41	35.93	30.10	35.39
Current density/3	38.44	36.06	34.97	36.62	36.52
Current density/4	39.28	38.85	35.10	37.23	**37.62**

a The boldface corresponds to the maximum value of the mean of the S/N ratios of a certain factor among the four levels.

In this study, an analysis of means (ANOM) was used to determine the optimal conditions. At first, the average S/N ratio of each factor was calculated at a certain level. For example, the averages S/N ratio of factor *I* at level *i* can be calculated from eqn (4).4
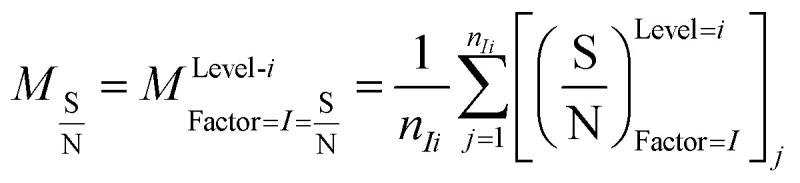


In eqn (4), *n*_*Ii*_ is the number of conditions for the factor *I* and level *i* in the experiment, which it is 2 in this study. Also, 
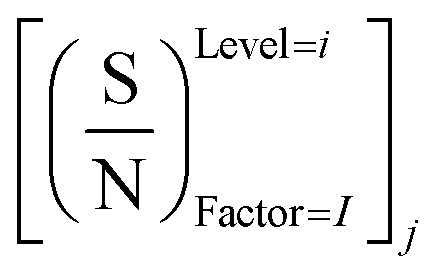
 is the S/N ratio of the experiments with the condition of factor *I* and level *i*. Similarly, this ratio is calculated for all factors and average levels. Finally, an experiment is conducted using the optimal conditions to confirm the method used. In this study, analysis of variance (ANOVA) was used to evaluate the effect of each factor on the rate of 2,4-D herbicide degradation. The percentage of the effect of each factor was determined using eqn (5).5
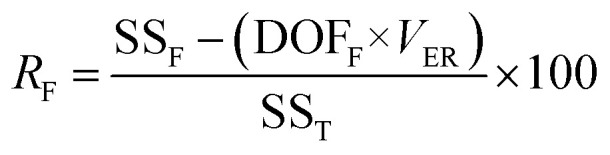


In this regard, DOF_F_ is the degree of freedom of each factor (one unit less than the number of levels of the target factor, which is 4 in this research). The total sum of squares (SS_T_) can also be calculated from eqn (6).6
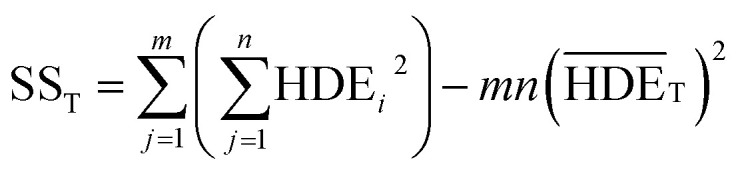


The value of 
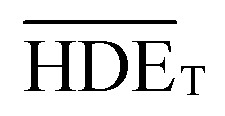
 is obtained from the eqn (7) in which *m* is the number of experiments (in this study is equal to 16) and *n* is the number of repetition of the experiment in the same conditions (it is equal to 2 in present study).7
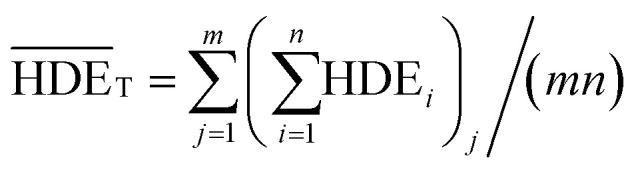


The sum of the factorial squares (SS_F_)^5^ is calculated using eqn (8).8
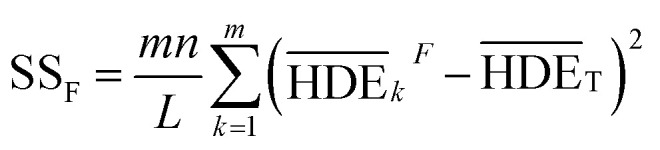

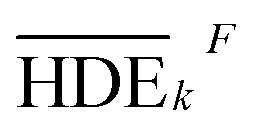
 is the mean of the measured values of the desired factor in the level of *k* and the error variance of *V*_ER_ is obtained from eqn (9).9
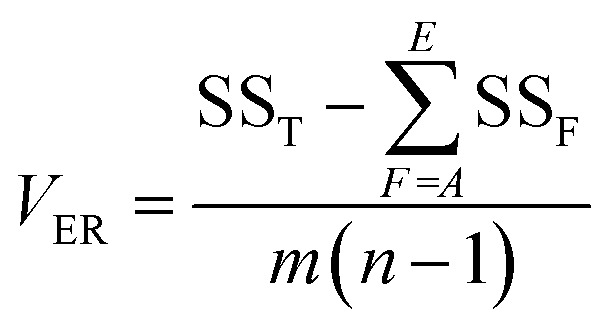


## 3. Results and discussion

### 3.1. Morphology of G/β-PbO_2_ electrode

The SEM technique was used to assess the microstructure of studied electrode under different magnifications and the obtained results were shown in Fig. S3(a).† These figure illustrated the agglomeration of the particles with limited individual particle boundary. The electrodeposited PbO_2_ microparticles on graphite show the crystalline structures β-PbO_2_ that β-PbO_2_ is the tetragonal structure.

Energy-dispersive X-rays (EDX) technique was utilized to determine the elemental structure of the β-PbO_2_ and its results were represented in Fig. S3(b).† These figure shows that the main elements existed in the β-PbO_2_ were the oxygen (O) and lead (Pb). Moreover, the weight percentage of oxygen (O) and lead (Pb) was observed to be 20.9% and 79.1%, respectively.

Furthermore, to discover the phases and crystallinity of PbO_2_ and purity of the deposited film, the X-ray diffraction patterns (XRD) was utilized. The XRD of the PbO_2_ layer deposited in the graphite interlayers is shown in Fig. S3(c)† in which the diffraction peaks of β form related to PbO_2_ has characterized. The XRD results are representative of the tetragonal structure of β-PbO_2_. The tetragonal β-PbO_2_, unlike the orthorhombic α-PbO_2,_ is associated with good conductivity,^34,35^ which can extraordinarily aid the electro-oxidation of contaminants in aqueous solution using anode studied. The major diffraction peaks were detected at 2*θ* of 25.4°, 32.0°, 36.2°, 49.1° and 62.5° for graphite electrode, which they corresponded to the (110), (101), (200), (211), (220) plane of β-PbO_2_, respectively.^17,36^ It is important to note that all samples demonstrate the existence of β-PbO_2_. In addition, Debye–Scherrer formula was applied to calculate the average size of β-PbO_2_;^17^ Considering the data calculated by this formula, the size of β-PbO_2_ crystals in the G/β-PbO_2_ electrode was 30.2 nm. These results are agreed with the results of SEM (Fig. S3a†).

### 3.2. The effect of supporting electrolyte

Supporting electrolyte (SE) is one of the most important parameters, which is effective on the electrochemical degradation process. In the present study, Na_2_SO_4_ was selected as an SE. The use of the Na_2_SO_4_, as an auxiliary electrolyte, to boost the electrical conductivity of the solution (it increases the current density), is led to indirect oxidization of by hydroxyl radicals and sulfate radicals during the electrolysis process,^37^ which it is agreed with the study conducted by Samarghandi *et al.* (2018); they have identified the Na_2_SO_4_ as best supporting electrolyte to degrade the 2,4-D.^14^ Moreover, Jaafarzadeh *et al.* (2018) have reported that, among the three supported electrolytes studied (Na_2_SO_4_, NaCl and NaNO_3_), the Na_2_SO_4_ is the best SE to degrade the 2,4-dichlorophenoxyacetic acid (2,4-D) herbicide,^38^ which it confirms the results of the present study. The optimum amount of Na_2_SO_4_ (used as supporting electrolyte, SE) was determined using the appropriate applied voltage. As shown in Table S2,† the required current densities can be obtained at all concentrations, but the higher potentials need to be applied at low SE dosages. Since the voltages were relatively similar in SE dosages of 1.0 and 1.5 g/250 cc, the SE dosage of 1.0 g/250 cc was accepted as the optimum value in this study (Table S2†) and the next experiments were conducted using the determined SE dosage.

### 3.3. Optimum conditions

In Table 1, the results are obtained and the S/N values for each of the related experiments are shown. Among the 16 designed experiments, the highest and lowest S/N values were related to the experiments 4 and 13, respectively and then the highest value obtained in Test 4 should be compared with the optimal mode. In Tables 4 and 5, the average analysis values for optimal conditions are observed. The highest values of *M* for each factor represent the optimal state of the agent. According to the optimum state tables for both 2D and 3D electrochemical processes, (1) pH level 2 (pH: 5), (2) time electrolysis level 4 (time: 100 min), (3) initial concentration of herbicide 2,4-D level 2 (*C*_0_: 100 mg L^−1^); and (4) current density level 4 (*j*: 9 mA cm^−2^) was obtained. The experiment was performed according to these conditions with two replications and the results were presented in Table 6. As shown in Table 6, the optimal conditions for Taguchi analysis are greater than S/N.

**Table 4:** The optimum conditions for 2,4-D herbicide degradation by 2D and 3D electrochemical process

Factor	*A*	*B*	*C*	*D*	HDE_1_	HDE_2_	S/N
Test 4 for 2D	3	100	200	9	72.71	71.2	37.14
Optimization condition for 2D	5	100	100	9	75.26	75.95	37.57
Test 4 for 3D	3	100	200	9	91.87	92.23	39.28
Optimization condition for 3D	5	100	100	9	93.68	93.31	39.42

**Table 5:** The average of the measurement results of a certain factor in the *k*th level and the average of total HDE for 2D and 3D electrodes

Electrodegradation process	Level	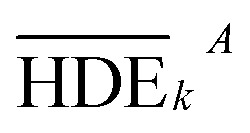	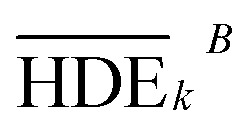	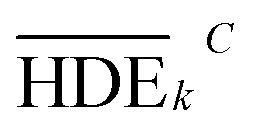	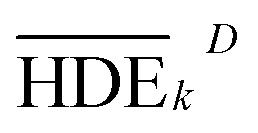	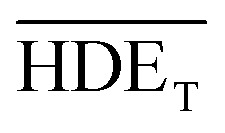
2D	Level 1	54.944	31.322	47.584	37.396	45.741
	Level 2	56	43.224	47.487	41.586	
	Level 3	39.404	52.108	45.346	47.127	
	Level 4	32.621	56.314	42.551	56.859	
3D	Level 1	75.651	51.942	68.317	58.254	66.435
	Level 2	76.759	63.969	68.19	62.419	
	Level 3	60.085	72.862	66.07	67.747	
	Level 4	53.244	76.965	63.158	77.319	

**Table 6:** Determination of percentage contribution of each factor

ED	Factor	DOF_F_	SS_F_	*R*_F_ (%)	SS_T_	*V*_ER_
2D	pH: *A*	3	3217.94	39.996	8026.02	2.61105
	Electrolysis time (min): *B*	3	2932.631	36.441		
	2,4-D concentration (mg L^−1^): *C*	3	134.242	1.575		
	Current density (mA cm^−2^): *D*	3	1699.43	21.076		
3D	pH: *A*	3	3246.81	40.435	8007.29	3.0106
	Electrolysis time (min): *B*	3	2946.47	36.684		
	2,4-D concentration (mg L^−1^): *C*	3	139.912	1.634		
	Current density (mA cm^−2^): *D*	3	1625.93	20.193		

The confirmation experiment was implemented based on the aforementioned optimum conditions, the HDE of 2D and 3D electrochemical process registered, and the S/N ratio was calculated (Table 4). The value of the S/N ratio under optimum conditions (2D = 37.57 and 3D = 39.42) slightly exceeds that in Test 4 (2D: 37.14 and 3D: 39.28), and the average HDE under optimum conditions (2D: 75.6% and 3D: 93.49%) indeed exceeds that in Test 4 (2D: 71.6% and 3D: 92.05%). Although the difference of the S/N ratio between the optimum conditions and Test 4 is very little, the 2,4-D herbicide concentration is substantially decreased from 200 mg L^−1^ (Test 4) to 100 mg L^−1^ (optimum conditions) and the pH is substantially increased from 3 (Test 4) to 5 (optimum conditions). Furthermore, the HDE increased from 71.6% to 75.6% for 2D and from 92.05% to 93.49% 3DThese results are pretty exciting due to the fact the lower 2,4-D herbicide and more pH corresponds to a better electrochemical process.

### 3.4. Effect of effective factors in electrochemical degradation process

In this study, the electro-degradation of 2,4-Dichlorophenoxyacetic acid (2,4-D) from aqueous solution was carried out using two and three-dimensional electrode (2D and 3D) reactor with graphite(G)/β-PbO_2_ anode. In oxidative degradation of organic compounds using PbO_2_ electrodes, the electro-generated physisorbed HO˙ on the surface of the electrode has a remarkable role in mineralization of the pollutants. The formation of adsorbed hydroxyl radical MO_*x*_(HO˙) by the oxidation of water molecules has introduced as the initial reaction in both kinds (active and non-active) of MO_*x*_ anodes, as shown in (eqn (10))^17,39^

10 MO_*x*_ + H_2_O → MO_*x*_(HO˙) + H^+^ + e^−^

In the next step, the electrochemically generated MO_*x*_(HO˙), which is accounted for as one of the strongest oxidants, mineralize the organic matter (eqn (11)).^17,39^ Additionally, the MO_*x*_(HO˙) can produce the O_2_ gas (eqn (12)); this reaction is considered as a competitor for the reaction represented in eqn (11).

11 R + MO_*x*_(HO˙) → *m*CO_2_ + *n*H_2_O + *x*H^+^ + *y*e^−^

12 2MO_*x*_(HO˙) → 2MO_*x*_ + O_2_ + 2H^+^ + 2e^−^

Previous studies revealed that the oxygen evolution reaction considerably depends on the value of oxygen evolution over-potential; so that, the oxygen evolution reaction has introduced as the main reaction for the electrodes with the low oxygen evolution over-potential; however, for the electrodes with high oxygen evolution over-potential, this reaction is difficult; hence, the reaction (11) occurs before the reaction (12), and it develops the efficiency of the mineralization reaction.^17,39^

**Fig. 1:**
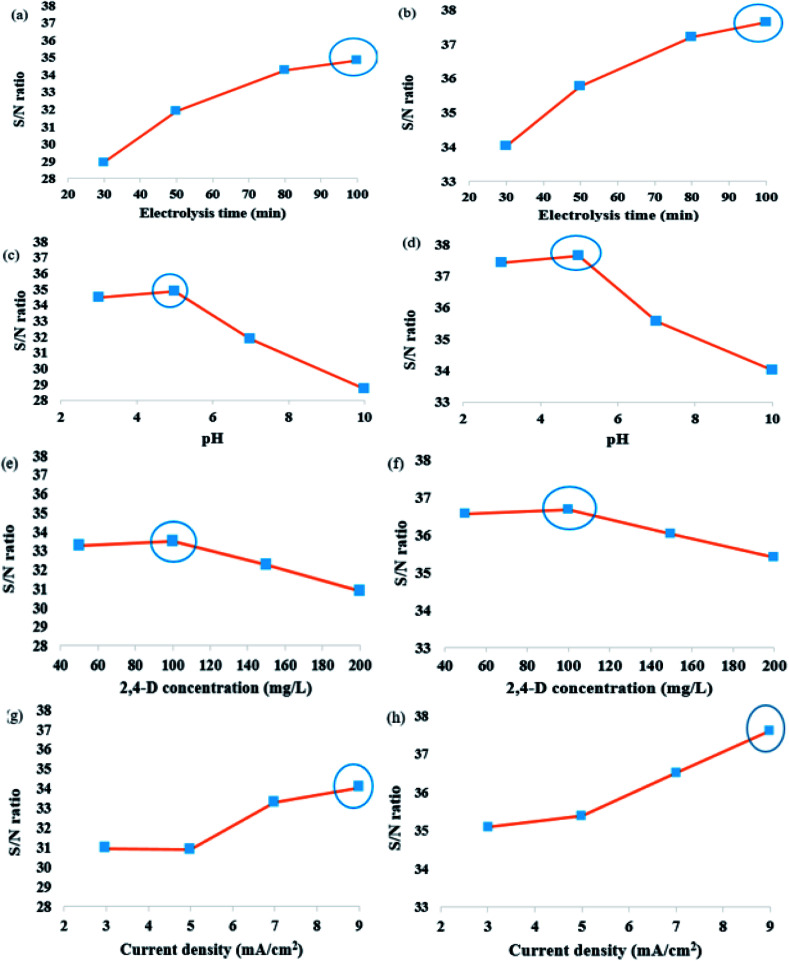
(a and b) The effect of electrolysis time, (c and d) the effect of pH, (e and f) the effect of 2,4-D concentration and (g and h) the effect of current density on the S/N ratio in the degradation of 2,4-D herbicide, a, c, e and g 2D and b, d, f and h 3D electrodegradation process. Circles on figures indicate optimum electrolysis time for electrochemical process.

The (*M*) are shown in (Fig. 1a–h) for the experimental conditions proposed by Taguchi method. Each of the factors affecting in 2,4-D herbicide degradation is as follows:

#### 3.4.1. Effect of electrolysis time

To understand the effect of electrolysis time on the 2,4-D degradation efficiency, the experiments were conducted using 2D and 3D electrode process at the electrolysis time in the range of 30–100 min. Generally, by increasing the electrolysis time, the degradation efficiency is developed and is reached a constant value at equilibrium state. The results based on S/N are shown in Fig. 1a and b. The obtained results showed that increasing the electrolysis time has also a direct effect on degradation efficiency, because the production of the radical hydroxyl is increased by increasing the electrolysis time and it is led to increasing the efficiency of 2,4-D herbicide degradation using 2D and 3D electrode process.

#### 3.4.2. Effect of pH of solution

The pH has been found as one of the most effective parameters on the electrochemical process. The effect of pH on the electrochemical process efficiency is explained by its effect on the electrically production of metal hydroxyls.^14^ The change in initial pH can lead to boost the degradation efficiency and to further optimize the treatment. The effect of pH on degradation of 2,4-D herbicide was assessed using various pH from 3–10. Fig. 1(c and d) shows the effect of pH on 2D and 3D electrode process with G/β-PbO_2_ anode. The results indicated that the highest 2,4-D degradation was attained at pH = 3 with S/N ratio of 34.84 for 2D electrode and 37.63 for 3D electrode process, respectively. At lower and higher pH values, the 2D and 3D electrode process efficiency was decreased. According to previous studies, the acidic pH values provide a better condition to increase the electrochemical process efficiency.^17,40,41^ Reducing the pH of the solution provides the situation to increase the production of hydroxyl radicals; since the potential of these radicals to degrade the pollutants is greater in the acidic environment, it will be resulted in increasing the oxidation efficiency.

#### 3.4.3. Effect of 2,4-D herbicide initial concentration

Fig. 1(e and f) shows that the efficiency of herbicide degradation is affected by the initial herbicide concentration; so that the degradation percentage decreases by increasing the concentration. The S/N for 2,4-D herbicide concentration decreases from 30.87 to 33.49 by increasing concentration from 100 to 200 mg L^−1^ using the 2D electrode process. It also indicates that the S/N for 2,4-D concentration decreases from 35.39 to 36.66 by increasing concentration from 100 to 200 mg L^−1^ using 3D electrode process. On the one hand, increasing the initial 2,4-D concentration increases its concentration on the electrode surface and, on the other hand, it leads to diminishing the electro-generated physisorbed HO˙ concentration by fouling the electrode surface by herbicide molecules and its intermediates^17,39^

#### 3.4.4. Effect of current density

In the electrochemical system, the current density (*j*) is accounted for as a crucial parameter because it influences the production of hydroxyl radical. To clarify the effect of *j* on the efficiency of 2D and 3D electrode process using the G/β-PbO_2_ anode for degradation of 2,4-D from aqueous solution, the experiments were conducted using the current densities in the range of 3–9 mA cm^−2^ and the results were shown in Fig. 1(g and h). The results of this figure, which were based on the value of S/N ratio, were indicative of a positive influence of *j* on the process performance, especially at higher *j* values. Based on the represented data in Fig. 1(g and h), an increase in the *j* from 3 to 9 mA cm^−2^ is led to increasing the S/N ratio from 30.89 to 34.03 and 35.1 to 37.62 using the 2D electrode and the 3D electrode, respectively. The optimum *j* was obtained to be 9 mA cm^−2^ and the higher 2,4-D degradation was at the higher *j*; which it is due to higher hydroxyl radical (·OH) generation at the higher *j*. The obtained results are agreed with the study conducted by Jaafarzadeh *et al.* (2018), who reported that increasing the current density increases the removal efficiency of herbicide.^38^

### 3.5. Percentage of contribution

Primarily, 
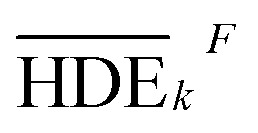
 (the average value of the measurement results of a certain factor in the *k*th level) was achieved from HDE_*i*_ in Table 1 and was represented in Table 5. By substituting 
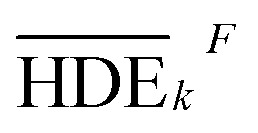
 and HDE_T_ (for 2D = 45.741 and for 3D = 66.435) into eqn (8), the factorial sum of squares, SS_F_, for each factor was individually calculated and was brought in Table 6. The total sum of squares, SS_T_, was determined using eqn (6). The variance of error, *V*_ER_, was achieved by substituting SS_F_ and SS_T_ (for 2D = 8026.02 and for 3D = 8007.29) in eqn (9). Finally, the percentage contribution of each factor, *R*_F_, was sequentially determined by the substitution of SS_F_, SS_T_, *V*_ER_ (for 2D = 2.61105 and for 3D = 3.0106), and DOF_F_ = 3 in eqn (5); and the obtained values were shown in Table 6. Considering their magnitudes, the rank order of the percentage contributions of each factor for 2D and 3D electro-degradation process is observed to be as follows: (1) the pH of solution (2D: 39.996% and 3D: 40.435%), (2) the electrolysis time (2D: 34.441% and 3D: 36.684%), (3) the herbicide initial concentration (2D: 1.575 and 2D: 1.634%) and (4) the current density (2D: 21.076% and 3D: 20.193%). Among the four factors, the pH was observed as the most influential factor on the electro-degradation. For instance, for 2,4-D initial concentration of (the least influential factor) of 200 mg L^−1^ in 3D electrode process, the average of herbicide degradation efficiency in Test 4, Test 7, Test 10, and Test 13 were 92.05, 72.53, 56.04, and 32.0%, respectively, and the average of all of them was 63.16%. In addition, for the 2,4-D initial concentration of 50 mg L^−1^, the average of herbicide degradation efficiency using the 3D electrode process in Test 1, Test 6, Test 10, and Test 14 were 55.25, 85.6, 56.04, and 40.49 respectively, and the average of all of them was obtained to be 59.34%. In other words, herbicide degradation efficiency in the initial concentration of 200 mg L^−1^ was 1.06 times higher than its degradation efficiency in initial concentration of 50 mg L^−1^, whereas herbicide degradation efficiency at the pH (the more influential factor) of 5 was about 2 times greater than the herbicide degradation efficiency at the pH = 10. So that, the average of herbicide degradation efficiency for pH 5 in Test 5, Test 6, Test 7, and Test 8 were 63.59, 87.6, 72.53, and 83.3%, respectively, and the average of all of them was achieved to be 77.7%. Furthermore, as the pH was 10, the average of herbicide degradation efficiency using the 3D electrode process in Test 13, Test 14, Test 15, and Test 16 were 32.0, 40.4, 72.7, and 67.7 respectively, and the average of all of them was 53.2%.

### 3.6. Comparison of degradation of 2,4-D herbicide using other methods

Reviewing the previous studies illustrated that several type of methods including electrochemical, electrochemical coagulation, coupled Fenton and biological oxidation and oxidation processes have evolved for the degradation of 2,4-D herbicide.^14,38,42–47^Table 7, compares these processes from the viewpoint of initial pH, electrolysis time, initial 2,4-D herbicide concentration, current density, electrode type, degradation efficiency and COD removal with the prepared electrode in this research. According to Table 7, our data in the most cases are superior to previously reported data.

**Table 7:** Comparison of degradation of 2,4-D herbicide using other methods^a^

Methods	Electrode type	pH	*C*_0_ (mg L^−1^)	Time (min)	*j*	Degradation (%)	COD (%)	Ref.
Electrochemical process	SS316	7	100	50	3 mA cm^−2^	17.65	*	7
Electrochemical process	Graphite	7	100	50	3 mA cm^−2^	47.76	*	7
Electrochemical process	Pb/PbO_2_	6	100	120	60 mA cm^−2^	57	56	2
Coupling electrooxidation and oxone	PbO_2_	4	40	90	30 mA cm^−2^	91	*	1
Photo assisted electro-peroxone	Platinum sheet	7	25	30	350 mA	89	*	3
Microwave activated electrochemical	BDD	*	100	180	55 mA	91	88	4
Coupled Fenton and biological oxidation	*	3	180	480	*	80	85–90	5
Oxidation process	*	3	200	120	*	68	*	6
Electrochemical coagulation process	iron	7	50	180	100 mA cm^−2^	91	*	8
Electrodegradation**	G/β-PbO_2_	5	100	100	9 mA cm^−2^	87.6	92.1	This study
Electrodegradation***	G/β-PbO_2_	5	100	100	9 mA cm^−2^	70.2	75.3	This study

a *Not considered, ** three-dimensional electrode and *** two-dimensional electrode.

### 3.7. Mechanism of 2,4-D herbicide degradation

**Fig. 2:**
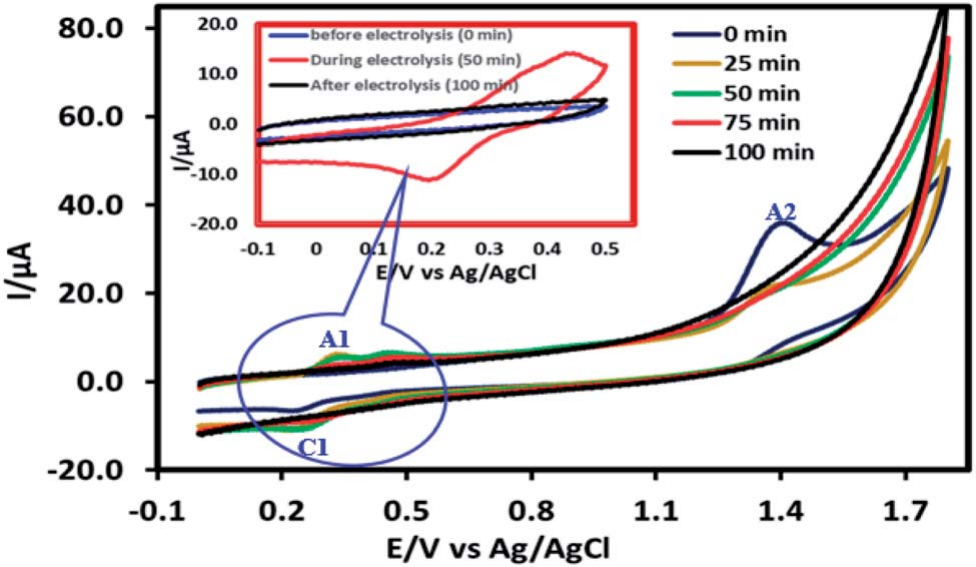
Degradation of 2,4-D herbicide solution before, during and after electrolysis at pH: 5 using G/β-PbO_2_ electrode in constant current electrolysis processes, that monitoring by cyclic voltammetry technique at 100 mV s^−1^ in 0.1 M Na_2_SO_4_ and 100 mg L^−1^ of 2, 4-D on a glassy carbon electrode.

**Fig. 3:**
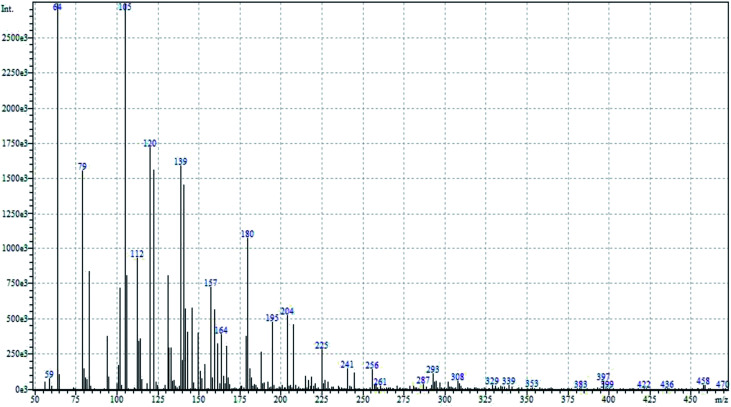
LC/MS chromatographs after degradation of 2,4-D herbicide at the optimum conditions.

**Scheme 1:**
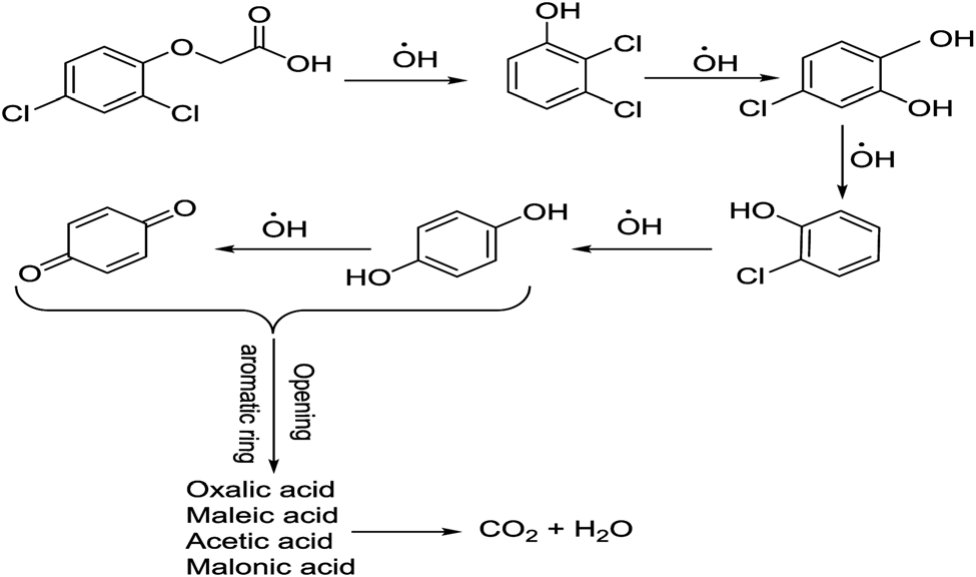
Proposed pathway for electrocatalytic degradation of 2,4-D herbicide by anodic oxidation on G/β-PbO_2_ electrode.

To determine the mechanism of electrochemical oxidation of 2,4-D herbicide, the electrolysis of this compound was carried out in 100 mg L^−1^ in a solution of electrolyte *M* Na_2_So_4_ 0.1 at pH = 5. This electrolysis was performed at a current density of 9 mA cm^−2^ for 100 min. The electrolysis process was followed up by a cyclic voltammetric technique. As shown in Fig. 2, during the electrolysis process, the peak flow of *A*_2_ is reduced and new peaks of *C*_1_ and *A*_1_ are created. Reducing the peak flow of *A*_2_ indicates the consumption of the herbicide and the appearance and increase of the new flow peaks of *C*_1_ and *A*_1_ represents the formation of the intermediates that are produced during the electrolysis process. As shown in Fig. 2, at the beginning of the electrolysis, the peak flow of *A*_2_ is reduced and the *A*_1_/*C*_1_ peaks begin to appear. This process continues until the *A*_2_ peak is completely removed. The complete removal of peaks of *C*_1_, *A*_1_ and *A*_2_ shows complete degradation of the 2,4-D herbicide. Considering our results and the results in the studies conducted by Souza *et al.* (2015),^48^ Jaafarzadeh *et al.* (2018),^38^ Fontmorin *et al.* (2012),^49^ the abovementioned mechanisms is offered for degradation of the studied herbicide. Moreover, in order to prove the proposed mechanism for degradation of 2,4-D herbicide and identification of intermediates, after 100 min, electrolysis was stopped and the LC-MS spectrum of the 2,4-D herbicide solution was provided. Fig. 3 shows the presence of molar mass for each of the intermediates in the corresponding spectrum, which it is accounted for as the reason for the correctness of pathway for the degradation of the 2,4-D herbicide and the proposed mechanism (Scheme 1). The analysis related to each intermediate is presented in Table 8.

**Table 8:** Identified products by LC-MS in the electrocatalytic degradation of 2,4-D herbicide by anodic oxidation on G/β-PbO_2_ electrode ([2,4-D]_0_ = 100 mg L^−1^, *j* = 9.0 mA cm^−2^, electrolysis time = 100 min and pH =5)

Molecular weight is observed (g mol^−1^)	Intermediate molecular structure
161–163	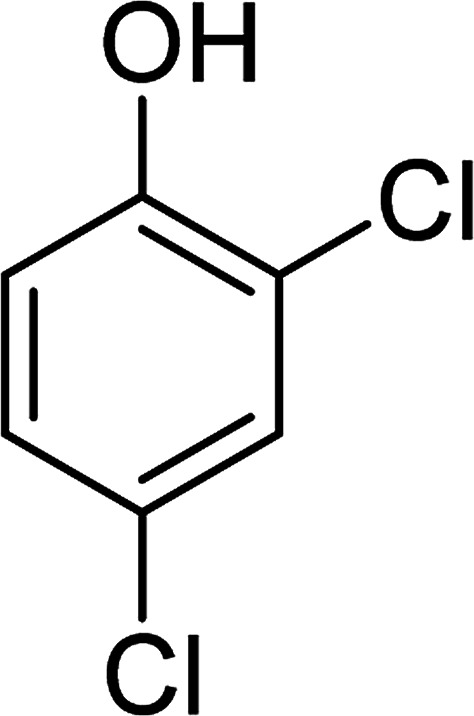
159	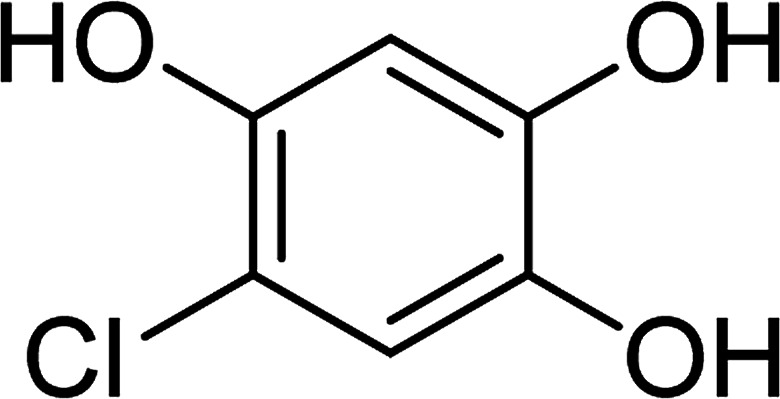
143–144	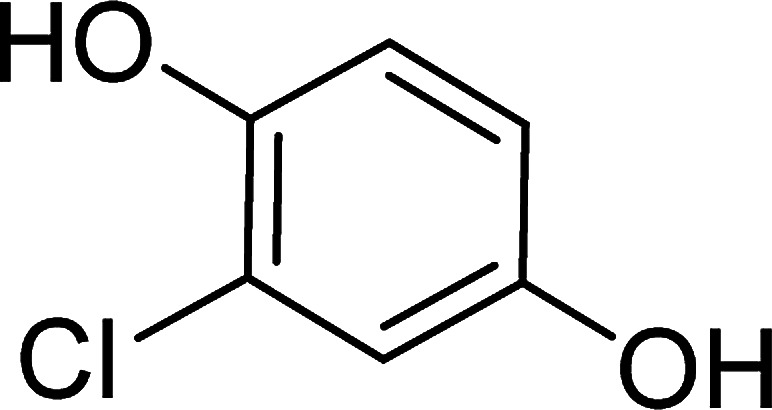
111–112	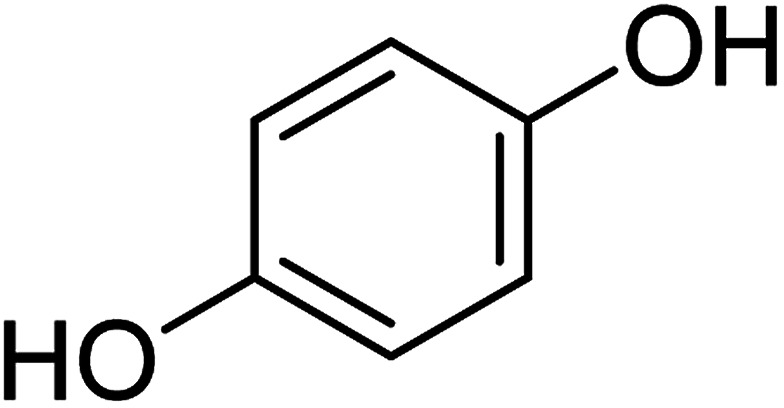
104–105	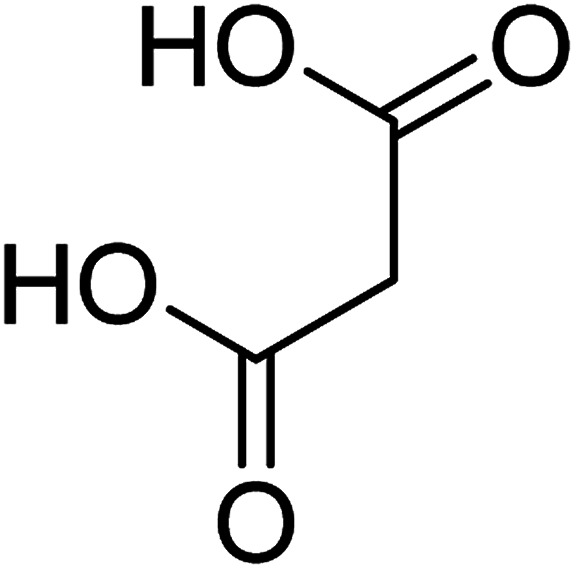
88–90	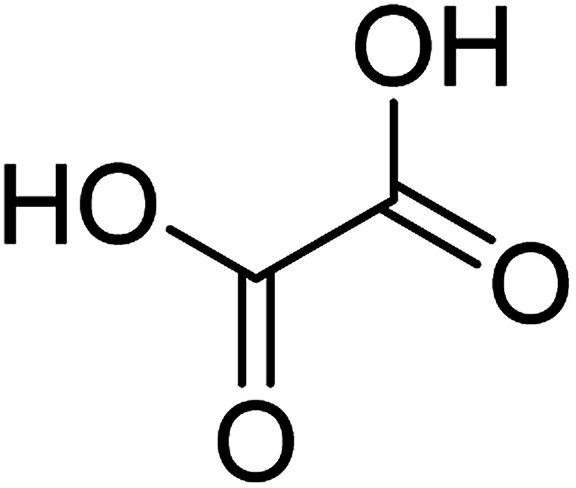
62–64	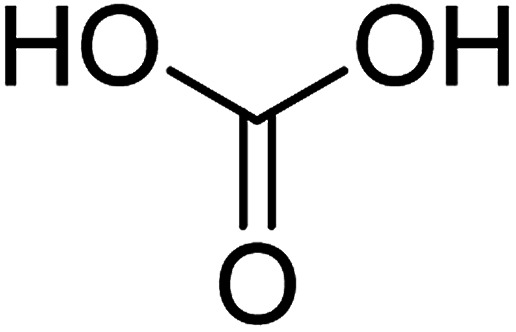

### 3.8. Kinetics studies

To compare the potential of 2D and 3D electrodes for the 2,4-D and COD removal efficiency, the rate constants (K) were calculated by plotting ln([2,4-D]_0_/[2,4-D]_*t*_) and ln([COD]_0_/[CODD]_*t*_) *versus* electrolysis time (*t*). For this purpose, the kinetics of the electrochemical degradation of herbicide 2,4-D and COD) was assessed under optimum conditions (electrolysis time: 100 min, pH: 5, Na_2_SO_4_: 1 g/250 cc and *j*: 9 mA cm^−2^.

**Fig. 4:**
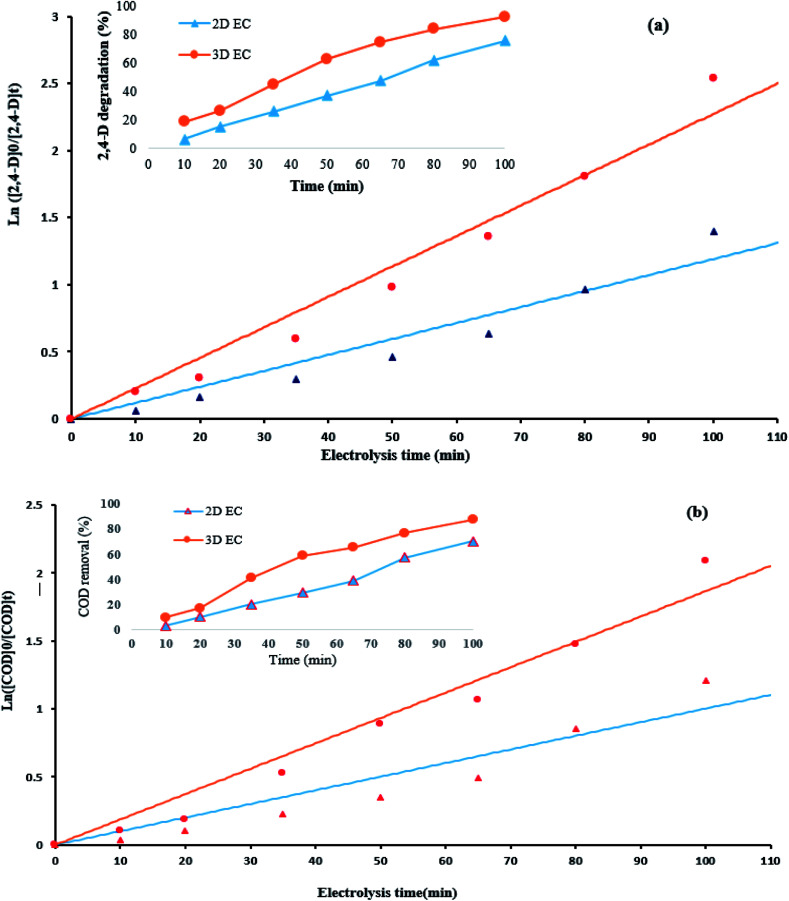
Kinetics of (a) 2,4-D degradation, (b) COD removal at the optimum conditions (herbicide initial concentration = 100 mg L^−1^, current density = 9 mA cm^−2^, pH = 5, Na_2_SO_4_ dosage = 1 g/250 cc).

The obtained plots with high correlation coefficients (*R*^2^) (Table S3†) imply that the kinetic behavior can be explained by a pseudo-first order model.^17,18,36,39^ The kinetics coefficients *K* are presented in Table S3.† The parameters of the kinetics (Table S3†) exhibited that there is a good linear relationship; furthermore, the degradation of 2,4-D on the G/β-PbO_2_ electrode followed pseudo-first order kinetics, which it indicates that this electrode has a good electro-degradation capability for the removal of 2,4-D. The rate constant (*k*) obtained for 2,4-D degradation using the 3D electrode (0.0228 min^−1^) was approximately 1.9 times higher than that of the traditional 2D electrode (0.0119 min^−1^). Moreover, the removal of COD on the G/β-PbO_2_ electrode followed pseudo-first order kinetics. The rate constant obtained for COD removal using the 3D electrode (0.0187 min^−1^) was observed to be approximately 1.8 times that of the traditional 2D electrode (0.0101 min^−1^). According to the results presented in Table S3,† the electrochemical degradation of the 2,4-D herbicide by the G/β-PbO_2_ electrode increased from 75.3 to 91.1% by an increase in the apparent rate constants from 0.0119 min^−1^ (2D process) to 0.0228 min^−1^ (3D process). In addition, COD removal efficiency increased from 70.2 to 87.6% by increasing the rate constants from 0.0101 (2D process) min^−1^ to 0.0187 min^−1^ (3D process), which it indicates the positive effect of the type of electrochemical process (2D and 3D) in degradation of 2,4-D and removal of COD (Fig. 4).

The higher degradation efficiency and COD removal efficiency using the G/β-PbO_2_ electrode can be related to the following reasons: (1) the presence of the high amount of coated PbO_2_ on graphite electrode that provides the greater sites for the production of hydroxyl radicals; (2) the lowest crystals size of β-PbO_2_ for G/β-PbO_2_ (Fig. S3†), which this is led to increasing the surface area, as a result, providing the high electro-generation efficiency of HO˙, which it offers better condition for degradation of pollutants.^17,39^ Furthermore, the half-life (*t*_1/2_) of herbicide degradation using 3D and 2D electrochemical was 31.5 and 58.2 min and its values for COD removal was 37.3 and 69.4 min, respectively (Table. S3†).

### 3.9. Leaching of lead ions

Leaching levels of lead ions were evaluated using ICP-OES after the completion of the 2,4-D degradation process using G/β-PbO_2_ electrode. According to results, after completion of the process, the Pb^2+^ concentration was observed to be 0.0013 ppm; the observed value was less than WHO guideline (0.01 mg L^−1^) for drinking water.^50^ This is indicative of the remarkable stability of the PbO_2_ coated on the surface of the electrodes, which it is consistent with the results of Ansari *et al.* (2018); in their study, the leaching level of lead in the solution after electrolysis with G/β-PbO_2_ electrode was reported to be 0.0035 mg L^−1^.^17^ The leaching of Pb^2+^ was also assessed using the cyclic voltammetry method; in this method, after electrolysis, 0.5 mg L^−1^ Pb(NO_3_)_2_ was added to the final solution and the related voltammogram was compared with the voltammogram of the final solution. Based on the Fig. S4,† the peak of the oxidation of the Pb^2+^ appeared at the potential of −0.5 V *vs.* Ag/AgCl, but such a peak is not observed in the voltammogram of the final solution. Considering the results, it can be concluded that the leaching of Pb^2+^ ions into the solution from the electrode surface is not occurred during electrolysis and after complete degradation of the herbicide.

## 4. Conclusion

In present study, the experimental design was carried out using the Taguchi method to determine the best condition for degradation of 2,4-D herbicide from aqueous solution using two and three-dimensional electrode (2D and 3D) reactor with G/β-PbO_2_ anode. According to percentage contribution of each factor, the pH of the solution was introduced as the most effective factor on the degradation of 2,4-D herbicide using 2D and 3D electrode (39.9% and 40.4%, respectively). In addition, the lowest effect on degradation efficiency was found to be related to initial 2,4-D concentration (1.5% and 1.6%, respectively). Optimum conditions in both electrochemical processes for degradation of 2,4-D was observed to be as follows: pH = 5, electrolysis time =100 min, current density = 9 mA cm^−2^ and 2,4-D initial concentration = 100 mg L^−1^. The kinetic studies revealed that the removal of COD and degradation of 2,4-D on the G/β-PbO_2_ electrode follows the pseudo-first order kinetic. Moreover, the Taguchi design was found to be a suitable method for optimizing the parameters. This method suggests a minimal number of experiments with high precision and accuracy and it has also the advantages such as reducing the cost and time of experimental investigation and, as a result, it reduces the chemical consumption in the optimizing the process. The results also showed that, after using the coated graphite, there were no harmful by-products and the 2,4-D herbicide was completely degraded to CO_2_ and H_2_O.

## Conflicts of interest

There are no conflicts to declare.

## Supplementary Material

RA-008-C8RA08471H-s001
