# Supplementary material for: Electrodegradation of 2,4-dichlorophenoxyacetic acid herbicide from aqueous solution using three-dimensional electrode reactor with G/β-PbO2 anode: Taguchi optimization and degradation mechanism determination
Source: RSC Adv. 2018 Nov 26;8(69):39256–68. doi: 10.1039/c8ra08471h (PMC9090970; doi:10.1039/c8ra08471h)
Supplement: RA-008-C8RA08471H-s001 [file RA-008-C8RA08471H-s001.pdf]

Electronic Supplementary Material (ESI) for RSC Advances.
This journal is © The Royal Society of Chemistry 2018

Supplementary data

Electrodegradation of 2,4-Dichlorophenoxyacetic acid from aqueous solution using three- dimensional electrode reactor with G/β-PbO2 anode: Taguchi Optimization and degradation mechanism determination

Chemicals

In this study, 2,4-Dichlorophenoxyacetic acid (2,4-D, chemical formula: $C_8H_6Cl_2O_3$, 99% purity), Lead nitrate ($Pb(NO_3)_2$, >99% purity), nitric acid (HNO_3, 95% purity), Granular activated carbon (GACs) and Sodium Sulfate (Na_2SO_4, 99% purity) were supplied from Sigma Aldrich (St. Louis, MO, USA). In addition, NaOH and HCl were purchased from Merck CO (Darmstadt, Germany). Double distilled water was applied to prepare the solution. 1N HCl and NaOH were applied to adjust the pH solution.

Preparation of the G/β-PbO_2 electrode

To prepare the G/β-PbO_2, the graphite electrodes (7.0 × 5.8 × 0.5 cm) were used. At the first, the soft sandpaper was utilized to polish the surface of the electrode. Then, it was truly washed by distilled water. The G/β-PbO_2 electrode was prepared through the electrochemical deposition on graphite. Initially, the graphite was sonicated with 40% NaOH solution and 1:1 (V/V) HNO_3/H_2SO_4 for 20 min. After that, the graphite was washed with deionized water and it was transferred to an electrochemical deposition cell containing 0.5 M $Pb(NO_3)_2$ in 0.1 M HNO_3 solution. The electrochemical deposition of PbO_2 on graphite was performed under the current density of 5 mA/cm^2 for 180-240 min. All of the experiments were carried out at 25 °C temperature (17,18).

Optimum Concentration of the Supporting Electrolyte (SE) Dose

The electrolyte has an important role in enhancing the solution conductivity and accelerating the electron transfer and, consequently, it can lead to increase the performance of the electrochemical reaction. Thus, it is necessary to determine the SE dose to advance the ionic strength, especially for the solutions without enough conductivity (18)). The optimum SE dose, to offer the studied current density (3-9 mA/cm^2), was determined by dissolving the concentrations of sodium sulfate (Na_2SO_4) (0.4-1.5 gr/250 cc) in 250 cc 2,4-D herbicide solution and the corresponding potential was then evaluated after applying the currents.

Table. S1: Controllable factors and their levels.

Factor	Description	Level 1	Level 2	Level 3	Level 4
**A**	pH	3	5	7	10
**B**	Electrolysis time (min)	30	50	80	100
**C**	2,4-D concentration (mg/L)	50	100	150	200
**D**	Current density (mA/cm^2	3	5	7	9

Table. S2. Supporting electrolyte (SE) dose providing desired currents and the corresponding potentials

Na_2SO_4 dose (gr/250 cc)	Current (A)	Voltage (V)	
		**2D**	**3D**
**0.4**	0.27	9.5	10.1
	0.45	13.8	14.6
	0.63	17.9	19.1
	0.81	22.5	23.5
**0.5**	0.27	7.9	9.4
	0.45	11.1	12.4
	0.63	15.3	16.2
	0.81	18.5	19.9
**0.8**	0.27	6.7	8.7
	0.45	8.6	9.8
	0.63	11.4	12.3
	0.81	13.5	14.9
**1**	0.27	5.9	7
	0.45	7.8	8.6
	0.63	9.2	10.9
	0.81	12.5	13.1
**1.2**	0.27	5.1	6
	0.45	6.5	7.9
	0.63	8.7	9.8
	0.81	10.9	11.7
**1.5**	0.27	4.8	5.5
	0.45	6.2	7.1
	0.63	7.5	8.6
	0.81	8.9	10.2

Table. S3. The kinetics for the electrochemical degradation of 2,4-D and removal of COD (time: 100 min, pH: 5, Na_2SO_4: 1gr/ 250 cc and *j*: 9 mA/cm^2)

**Electrochemical process**	**k (min^{-1})**		**R^2**		**$t_{1/2}$ (min)**	
	2,4-D	COD	2,4-D	COD	2,4-D	COD
**2D EC**	0.0119	0.0101	0.935	0.911	58.2	69.4
**3D EC**	0.0228	0.0187	0.988	0.975	31.5	37.3

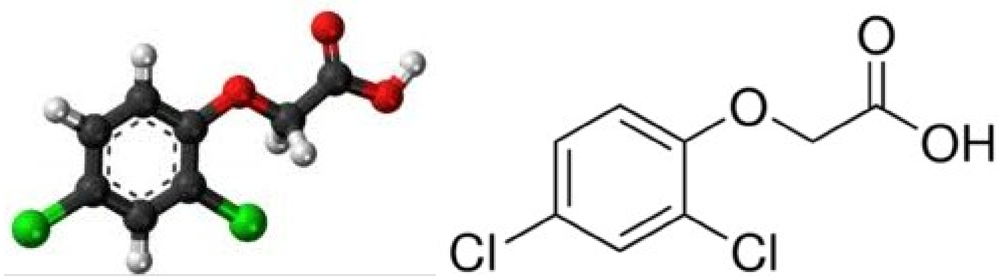

Fig. S1. Chemical structures of 2.4-Dichlorophenoxyacetic acid herbicide

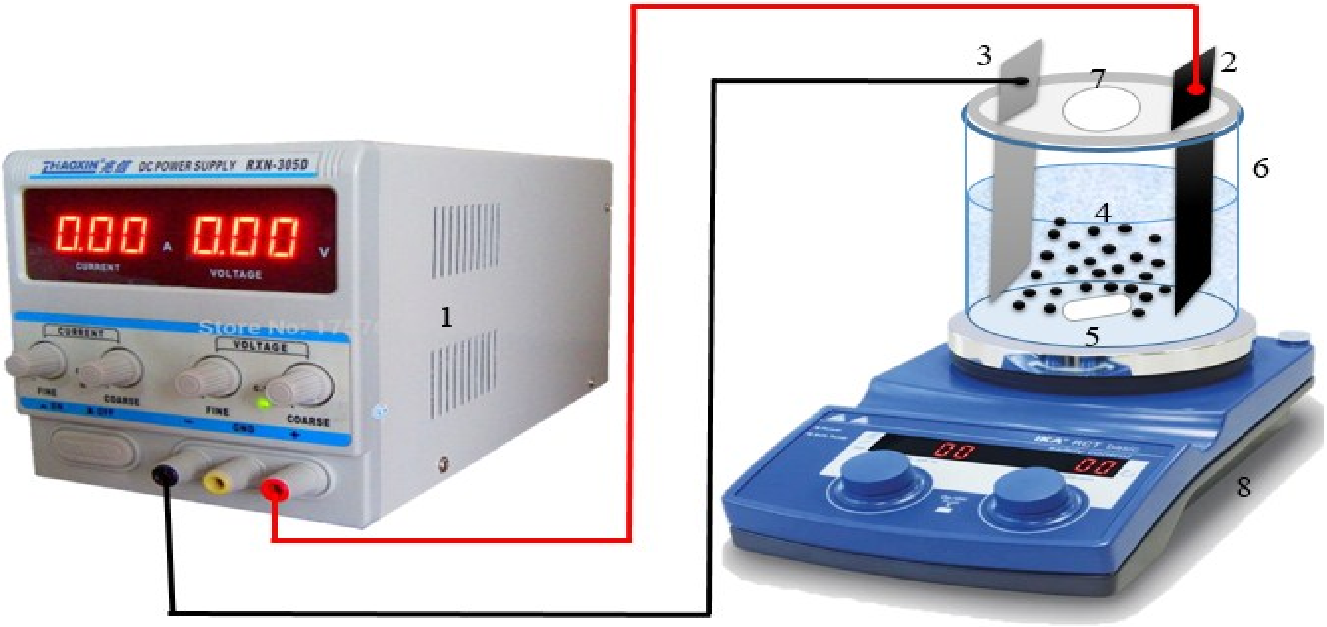

Fig. S2. The batch electrochemical reactor, 1. DC Power supply, 2. Anode electrode (G/β-PbO_2), 3. Cathode electrode (SS316), 4. Simulated 2,4-D solution (including GACs), 5. Magnetic- bar stirrer, 6. Electrochemical cell, 7. Sampling port and 8. Magnetic stirrer controlled

(a)

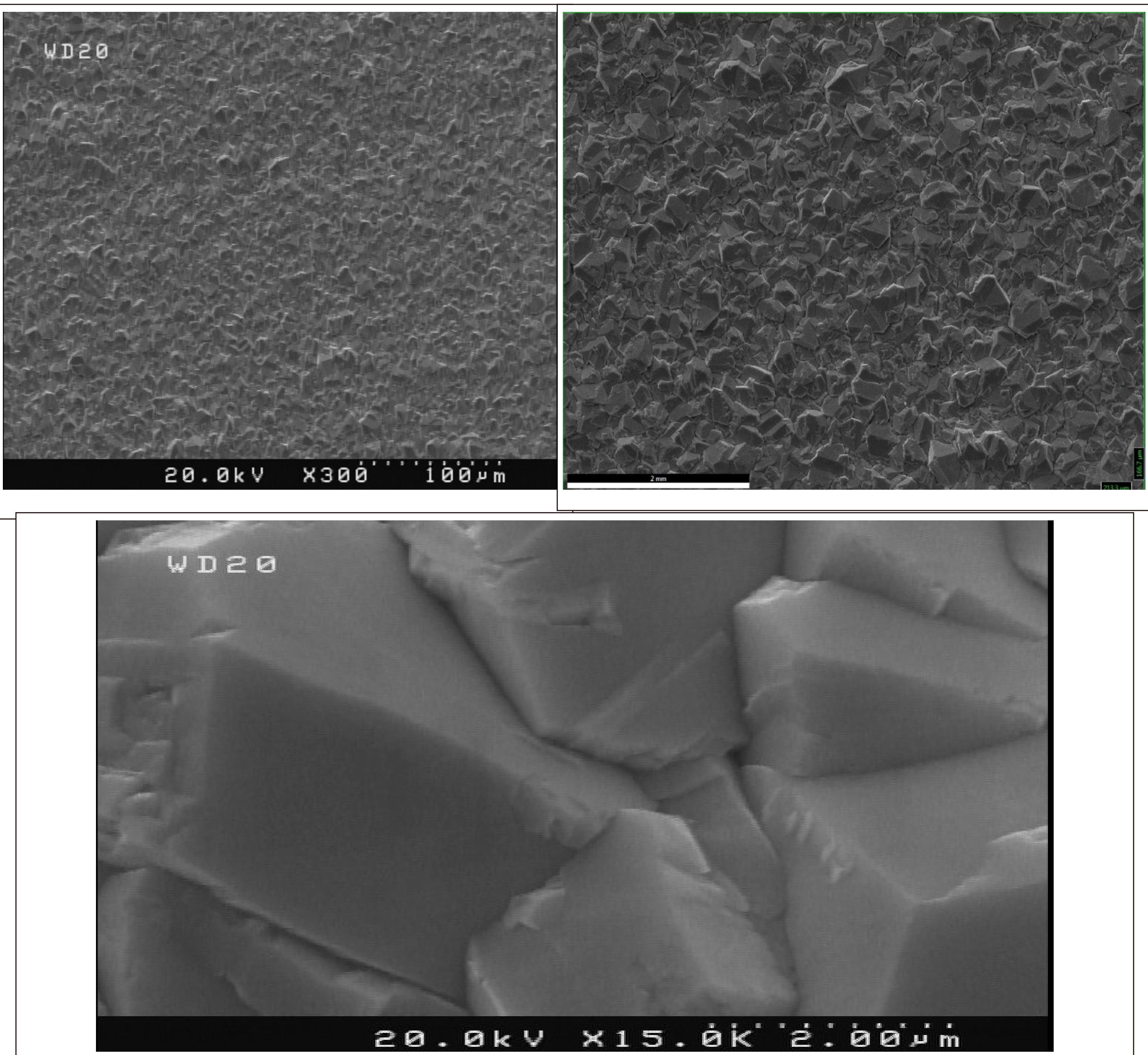

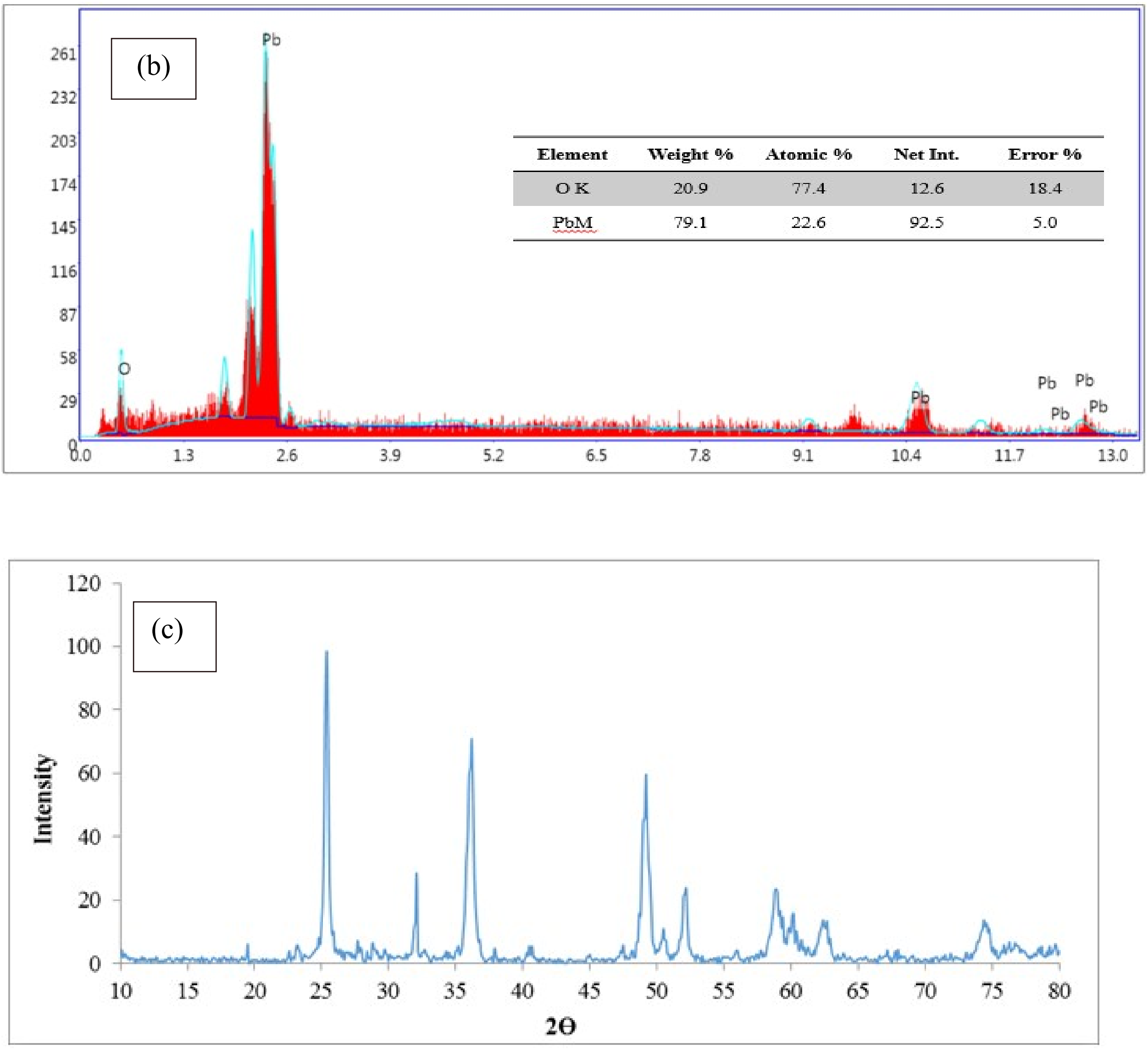

Fig. S3. (a) SEM micrographs of the surface of G/β-PbO_2 at different magnifications; (b) EDX analysis of G/β-PbO_2 anode; (c) XRD analysis of G/β-PbO_2 anode.

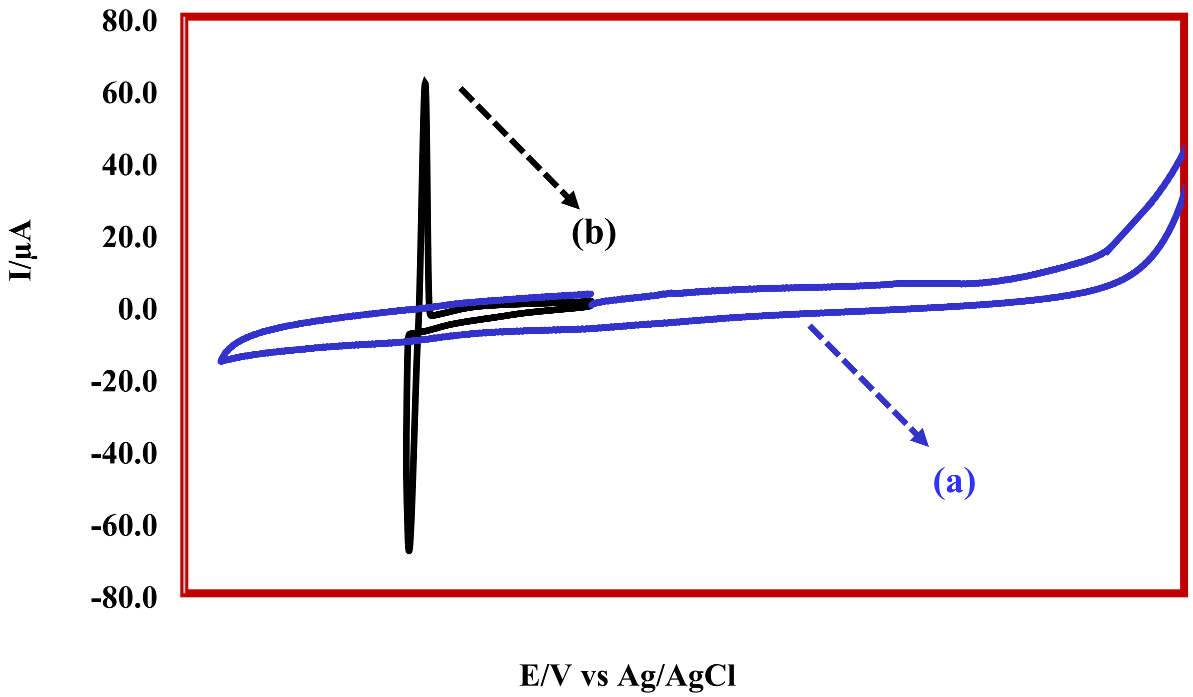

Fig. S4. Cyclic voltammogram of the electrolyzed solution containing 2,4-D herbicide + Na_2SO_4. (a) before spiked with $Pb(NO_3)2$ and (b) after spiked with $Pb(NO_3)2$ (1.5×10^{-6} M), at glassy carbon electrode, in aqueous solution, pH = 5.0. Scan rate: 100 mV/s at room temperature.
